# Supplementary material for: Comparative Genomics Reveals Multipartite Genomes Undergoing Loss in the Fungal Endosymbiotic Genus Mycetohabitans
Source: Genome Biol Evol. 2025 Dec 1;17(12):evaf231. doi: 10.1093/gbe/evaf231 (PMC12696581; doi:10.1093/gbe/evaf231)
Supplement: evaf231_Supplementary_Data [file evaf231_supplementary_data.zip › Supplementary Material.pdf]

Comparative Genomics of Mycetohabitans - Supplementary Information

Table S1. All long-read sequenced Mycetohabitans genome assemblies used in this study.

Host Fungal Species	Host Fungal Accession	Bacterial Species	Bacterial Strain	Collection Location	Bacterial GenBank Accession
Long read PacBio Sequencing (Carpenter, et al, 2024)					
Rhizopus microsporus	CBS111563	*M. endofungorum*	B3<br>HKI 0455	Vietnam	CP062180–CP062181
Rhizopus microsporus	ATCC52813	*M. endofungorum*	B13<br>HKI 0402	Ukraine	CP132744–CP132745
Rhizopus microsporus	CBS112285	*Mycetohabitans endofungorum*	B5<br>HKI 0456	Mozam-bique	CP062178–CP062179
Rhizopus microsporus	ATCC52814	*Mycetohabitans endofungorum*	B14<br>HKI 0403	Georgia	CP132741–CP132743
Rhizopus microsporus	ATCC52812	*Mycetohabitans rhizoxinica*	B12	USA	CP062175–CP062177
Rhizopus microsporus	NRRL5546	*Mycetohabitans sp.*	B46	Brazil	JADBGL000000000
Rhizopus microsporus	NRRL5547	*Mycetohabitans rhizoxinica*	B47	Philippines	CP062173–CP062174
Rhizopus microsporus	NRRL5549	*Mycetohabitans rhizoxinica*	B49	Wisconsin, USA	CP062171–CP062172
Rhizopus microsporus	NRRL5560	*Mycetohabitans endofungorum*	B60	USA	CP062168–CP062170
Long read ONT sequencing – This Study					
Rhizopus microsporus	ATCC62417	*Mycetohabitans rhizoxinica*	B1<br>HKI 0454	Japan	CP184277-CP184279
Rhizopus sp. strain F-1360.	ATCC20577	*Mycetohabitans rhizoxinica*	B2<br>HKI 0512	Japan	CP176780-CP176782
Rhizopus oryzae	NRRL62023	*Mycetohabitans endofungorum*	B23	Nebraska, USA	CP184774-CP184776
Rhizopus microsporus	NRRL5551	*Mycetohabitans endofungorum*	B51	Philippines	CP173333-CP173335
Rhizopus liquefaciens (Rhizopus oryzae)	NRRL 1514	*Mycetohabitans rhizoxinica*	B514	Unknown	CP176773-CP176775
Rhizopus microsporus	NRRL5553	*Mycetohabitans endofungorum*	B53	South Africa	CP173328-CP173330
Rhizopus microsporus van Tieghem	NRRL6255	*Mycetohabitans rhizoxinica*	B55	Ohio, USA	CP173325-CP173327
Rhizopus microsporus	NRRL5558	*Mycetohabitans rhizoxinica*	B58	USA	CP184771-CP184773
Rhizopus americanus	NRRL66675	*Mycetohabitans endofungorum*	B75	Pakistan	CP173322-CP173324
Rhizopus microsporus	CBS 308.87	*Mycetohabitans sp.*	B8<br>HKI 0404	Victoria, Australia	CP173344-CP173345

Rhizopus microsporus var. oligosporus	NRRL 2710	*Mycetohabitans rhizoxinica*	B10	Indonesia	CP176778-CP176779
Rhizopus oligosporus	NRRL 6203	*Mycetohabitans rhizoxinica*	B203	Djakarta, Indonesia	CP176776-CP176777
Rhizopus microsporus	NRRL 13129	*Mycetohabitans endofungorum*	B29	Unknown	CP173342-CP173343
Rhizopus sp.	NRRL2934	*Mycetohabitans rhizoxinica*	B34	California, USA	CP173340-CP173341
Rhizopus microsporus	NRRL5546	*Mycetohabitans sp.*	B46	Brazil	CP173320-CP173321
Rhizopus microsporus	NRRL5548	*Mycetohabitans rhizoxinica*	B48	Illinois, USA	CP173338-CP173339
Rhizopus microsporus	NRRL5550	*Mycetohabitans rhizoxinica*	B50	New York, USA	CP173336-CP173337
Rhizopus microsporus	NRRL5552	*Mycetohabitans rhizoxinica*	B52	Ohio, USA	CP173331-CP173332
Rhizopus microsporus var oligosporus	CBS 339.62	*Mycetohabitans rhizoxinica*	B62	Indonesia	CP178341-CP178342
Rhizopus arrhizus	NRRL2582	*Mycetohabitans rhizoxinica*	B82	Ohio, USA	CP184791-CP184792

Table S2. Guanine-cytosine percent (%GC) for each replicon, and the differences between chromosome and chromid and plasmid respectively.

Strain	Species*	%GC			%GC difference	
		Chromosome	Chromid	Plasmid	Chromosome – Chromid	Chromosome-Plasmid
**B10**	Mrh	61.484	59.758	-	1.7260	-
**B12**	Mrh	61.270	59.575	56.804	1.6997	4.470
**B13**	Mef	60.924	59.559	-	1.3644	-
**B14**	Mef	61.037	59.997	56.263	1.0397	4.774
**B1**	Mrh	61.213	59.705	57.429	1.5074	3.783
**B203**	Mrh	61.484	59.758	-	1.7260	-
**B23**	Mrh	61.254	59.331	58.424	1.9230	2.830
**B29**	Mef	61.583	60.017	-	1.5661	-
**B2**	Mrh	61.267	59.223	56.937	2.0437	4.329
**B34**	Mrh	61.308	59.932	-	1.3766	-
**B3**	Mef	60.987	59.467	-	1.5209	-
**B46**	Msp	61.445	59.579	-	1.865	-
**B47**	Mrh	61.480	59.769	-	1.7105	-
**B48**	Mrh	61.374	59.461	-	1.912	-
**B49**	Mrh	61.312	59.701	-	1.6117	-
**B50**	Mrh	61.312	59.703	-	1.6088	-
**B514**	Mrh	61.267	59.224	56.939	2.043	4.328

**B51**	Mef	61.396	59.655	57.262	1.7403	4.133
**B52**	Mrh	61.353	59.498	-	1.8551	-
**B53**	Mef	61.191	60.149	56.615	1.0417	4.575
**B55**	Mrh	61.540	59.630	55.863	1.9106	5.676
**B58**	Mrh	61.303	59.790	55.250	1.5133	6.053
**B5**	Mef	61.583	60.016	-	1.5663	-
**B60**	Mef	61.189	60.282	56.641	0.9070	4.547
**B62**	Mrh	61.483	59.758	-	1.725	-
**B75**	Mef	61.252	59.589	57.618	1.6630	3.63
**B82**	Mrh	61.645	58.948	-	2.6970	-
**B8**	Msp	61.396	59.932	-	1.4633	-

Mycetohabitans endofungorum, Mef; *Mycetohabitans rhizoxinica*, Mrh; *Mycetohabitans* sp., Msp

Table S3. MOBTyper output on plasmid sequences (separate file)

Table S4. Reference genomes from *Burkholderia* sensu lato genera that were used in Average Nucleotide Identity analysis for Figure 1.

NCBI ACCESSION	SPECIES
NZ_CP012981.1	Burkholderia cepacia ATCC 25416 strain UCB 717
NZ_JFHC01000001.1	Caballeronia glathei strain DSM 50014 contig1
NZ_CP047396.1	Candidatus Vallotia cooleyia isolate 19-005_Vallotia chromosome
NZ_CP047399.1	Candidatus Vallotia lariciata isolate Ad13-081_Vallotia chromosome
NZ_CP080504.1	Candidatus Vallotia sp. (ex Adelges kitamiensis) isolate Adelges kitamiensis 18-608 chromosome
NZ_OU343031.1	Candidatus Vallotia tarda isolate MYVALT chromosome 1
NZ_ABLD01000070.1	Paraburkholderia graminis C4D1M
NZ_RBZU01000001.1	Pararobbsia silviterrae strain DHC34 scaffold1
NZ_LAQU01000001.1	Robbsia andropogonis strain ICMP2807 contig00001
NZ_PTIR01000001.1	Trinickia symbiotica strain JPY-345 Ga0139055_101

Table S5. Syny output, shared genes across *M. rhizoxinica* B1, *M. endofungorum* B5, *Mycetohabitans* spp. B8 and B46 genomes. (separate file)
Table S6. Anvi'o pangenome gene cluster summaries for whole genome pangenome analyses. (separate file)

Table S7. Percentage of genome masked by transposable element (%TE) and percentage of total coding DNA sequences identified as putative pseudogenes for each strain.

Species*	Strain	Plasmid presence	%TE	%Total pseudogene
Mef	B75	1	6.36	9.86046512
Mef	B60	1	4.53	7.68983269
Mef	B53	1	4.44	8.21012897
Mrh	B52	0	4.25	8.57409133
Mef	B51	1	4.05	9.5209759
Mrh	B82	0	3.86	8.63607393
Mrh	B12	1	3.39	8.35380835
Mef	B3	0	3.33	8.09286899
Mef	B14	1	3.12	9.95085995
Mrh	B1	1	3.08	8.91544118
Mrh	B2	0	3.01	8.74365861
Mrh	B514	1	3.01	8.77873992
Mrh	B10	0	2.79	7.90866976
Mrh	B203	0	2.79	7.90866976
Mrh	B62	0	2.79	7.87557909
Mrh	B34	0	2.65	8.58876118
Mef	B13	0	2.64	9.96394625
Mrh	B58	1	2.59	8.82533825
Mrh	B23	1	2.43	8.8762984
Msp	B8	0	2.31	8.84562842
Mrh	B49	0	2.12	6.84976837
Mrh	B50	0	2.11	7.86407767
Mrh	B47	1	2.1	6.37516689
Mef	B29	0	1.91	8.04480652
Mef	B5	0	1.91	8.1043956
Msp	B46	0	1.78	7.58550627
Mrh	B55	1	1.73	8.22097994
Mrh	B48	0	1.58	7.82219159
B_cepacia_ATCC_25416		-	0.59	1.51669641
C_glathei_DSM_50014		-	0.79	3.1949279
Ca_V_lariciata_Ad13_081		-	0.18	5.87449933
Ca_V_sp_18_608		-	0	4.63078849
P_graminis_C4D1M_ctg63		-	0.76	3.62275449
P_silviterrae_DHC34		-	0.24	1.44442487
T_symbiotica_JPY_345		-	0.33	-
R_andropogonis		-	1.43	-

**Mycetohabitans endofungorum*, Mef; *Mycetohabitans rhizoxinica*, Mrh; *Mycetohabitans* sp., Msp. “-“ indicates that the field was not examined.

Table S8. Biosynthetic gene cluster summary. (separate file)

Supplementary Figures

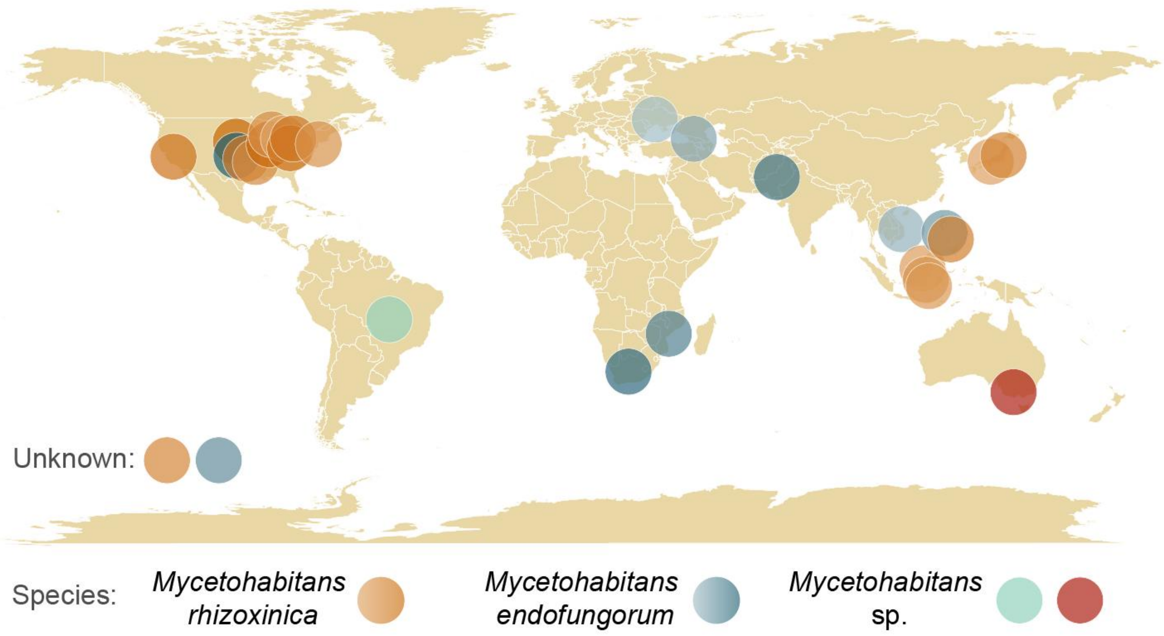

Figure S1. Global map showing isolation country for each fungal host of the *Mycetohabitans* strains used in this study. The species of the bacterial symbionts are indicated by color, with variation in orange and blue corresponding to strain relationship in Figure 1A, i.e. strains with similarly dark orange or blue are most closely related.

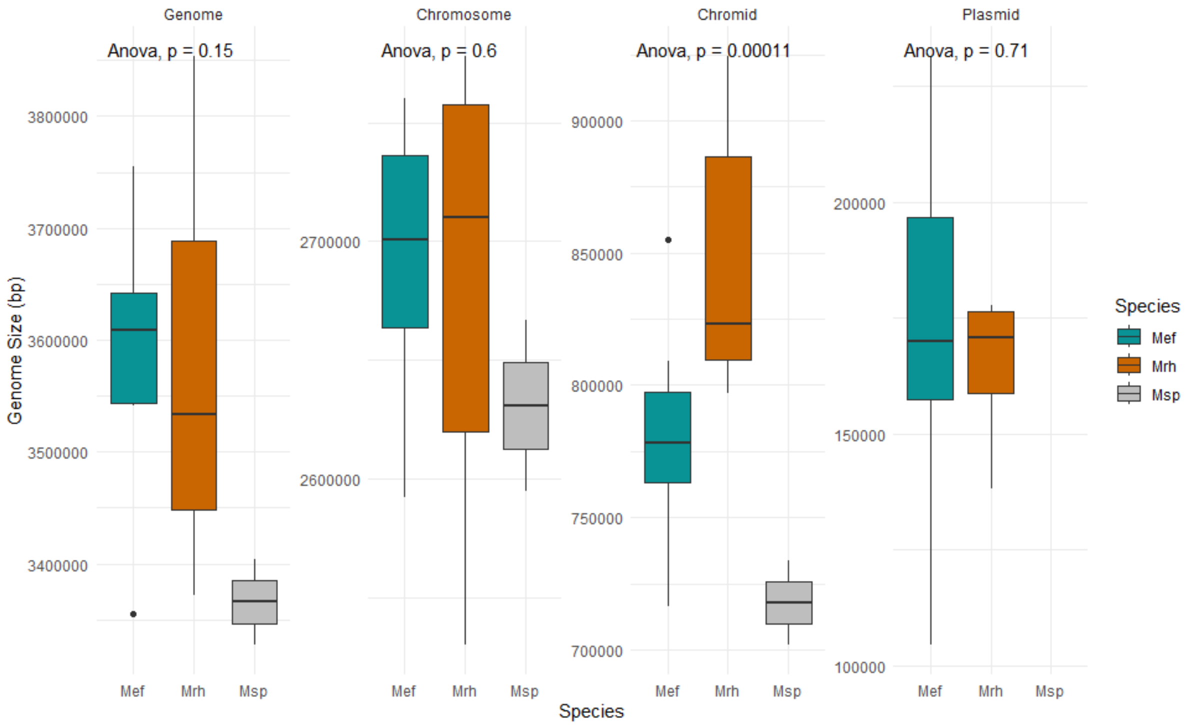

Figure S2. Distribution of genome and replicon sizes across all *Mycetohabitans* strains. Msp, *Mycetohabitans* spp.; Mrh, *M. rhizoxinica*; Mef, *M. endofungorum*. Means comparison was done by anova and significance value is indicated; only chromid size was significantly different by species.

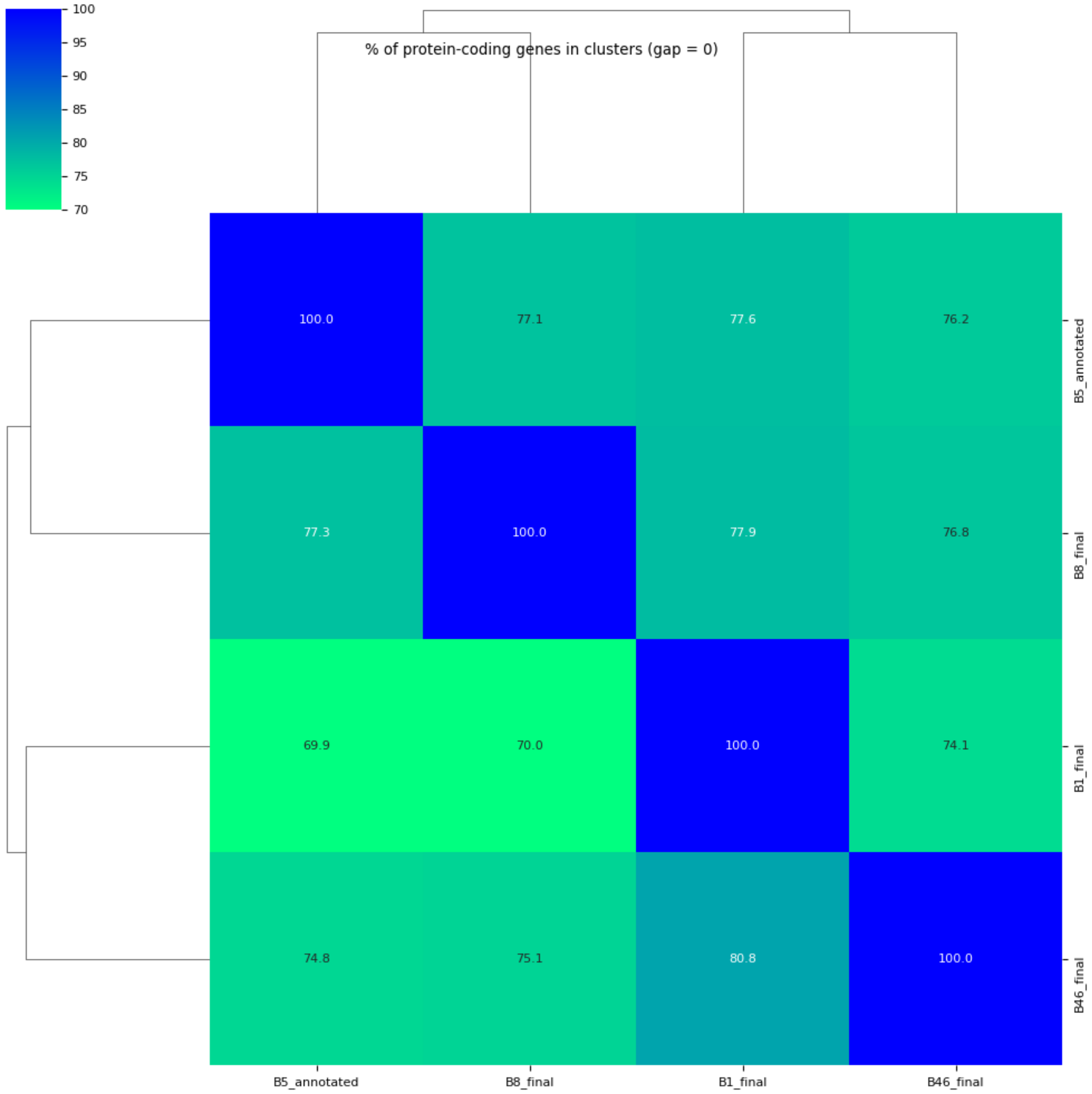

Figure S3. Heatmap showing hierarchical percent collinearity in genome based on protein clusters (evalue = 1e-10).

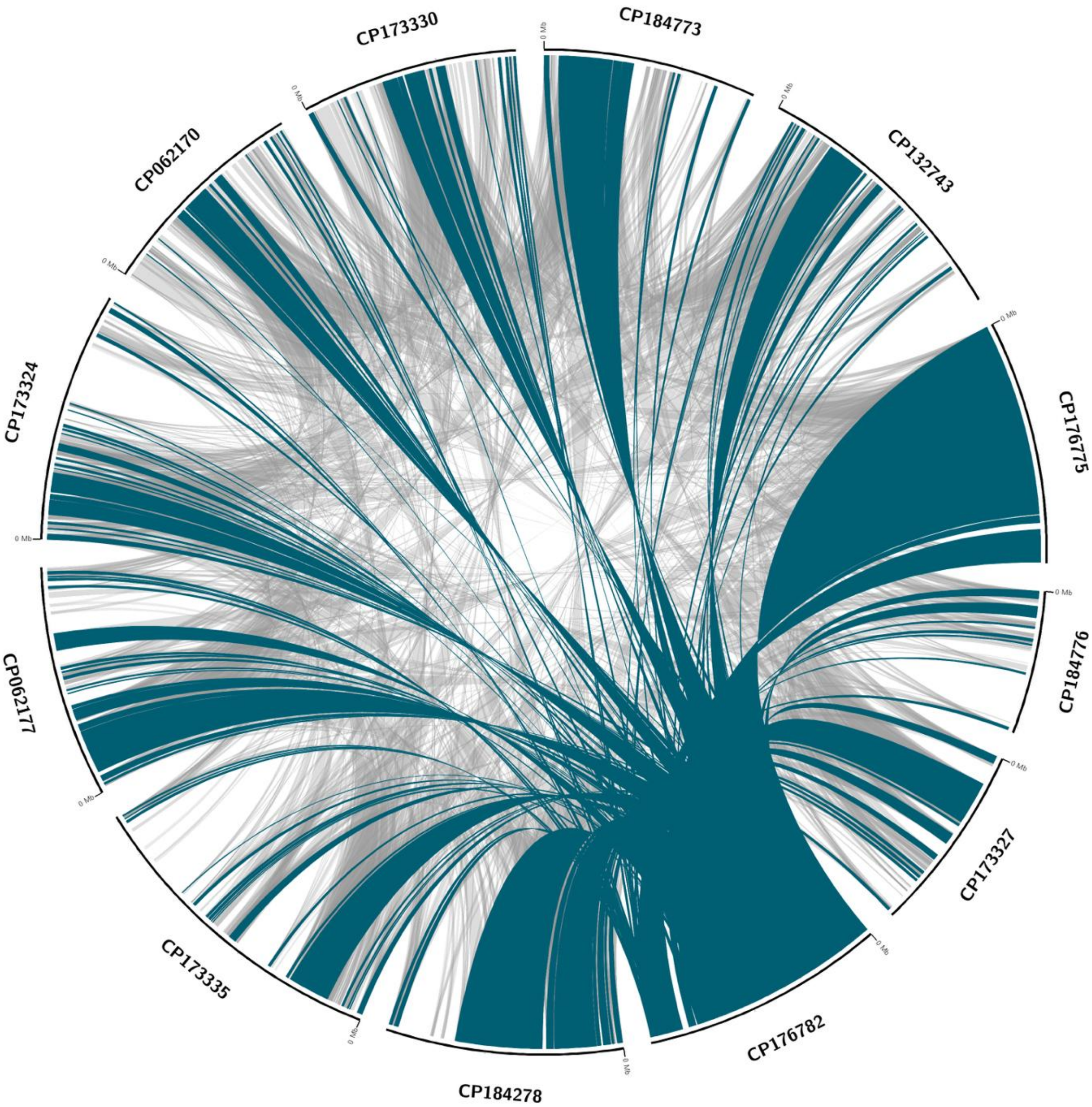

Figure S4. Syntenic relationship overview across *Mycetohabitans* plasmids. IDs beginning with CP correspond to NCBI sequence accessions. Teal lines indicate comparison to strain B2 as a reference.

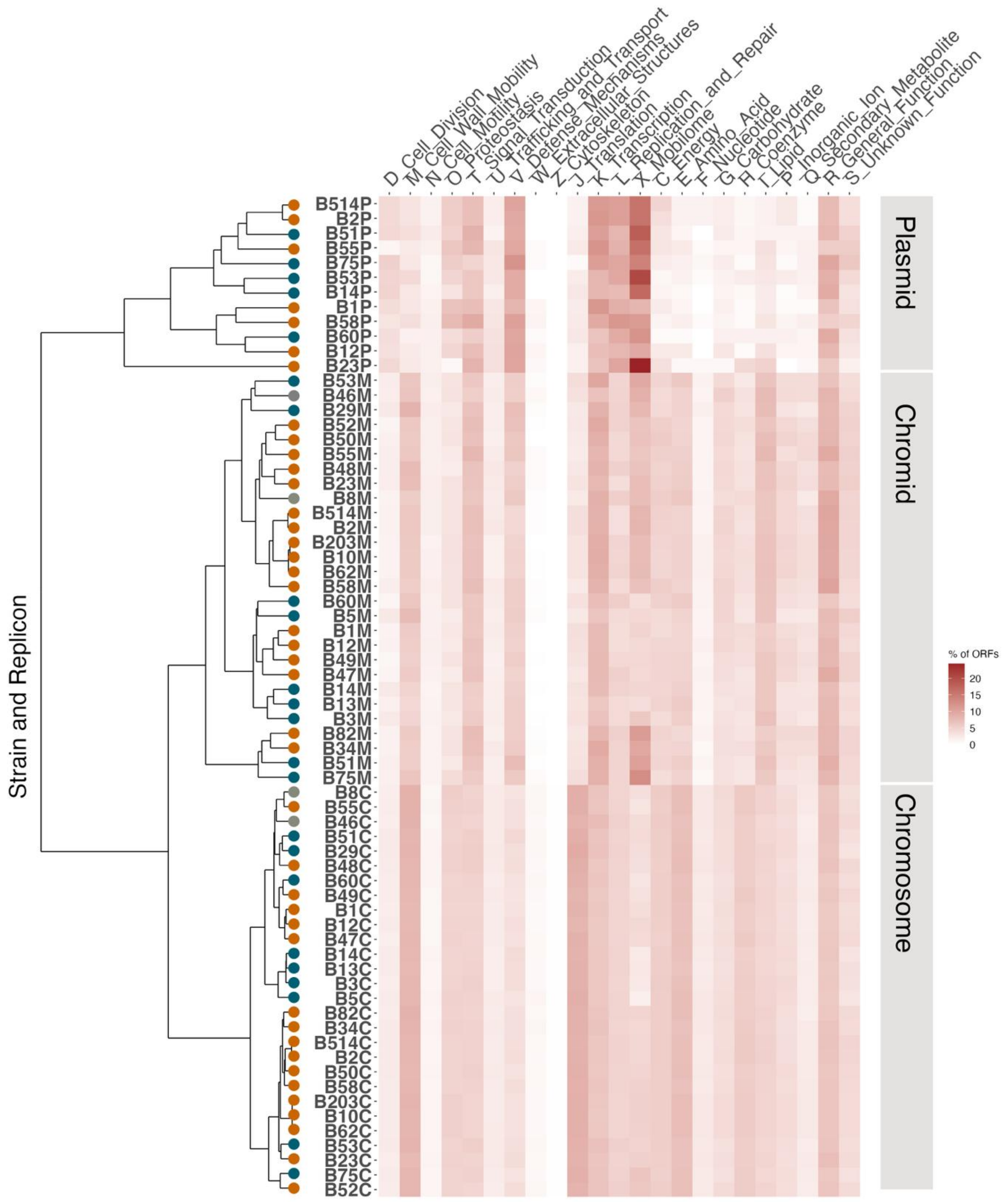

Figure S5. Distribution of COG gene groups distribution across replicons present in *Mycetohabitans* strains. Orange dots on the dendrogram indicate *M. rhizoxinica,* blue dots indicate *M. endofungorum*, and grey dots indicate *Mycetohabitans* sp. Chromosome, C; Chromid, M; Plasmid, P.

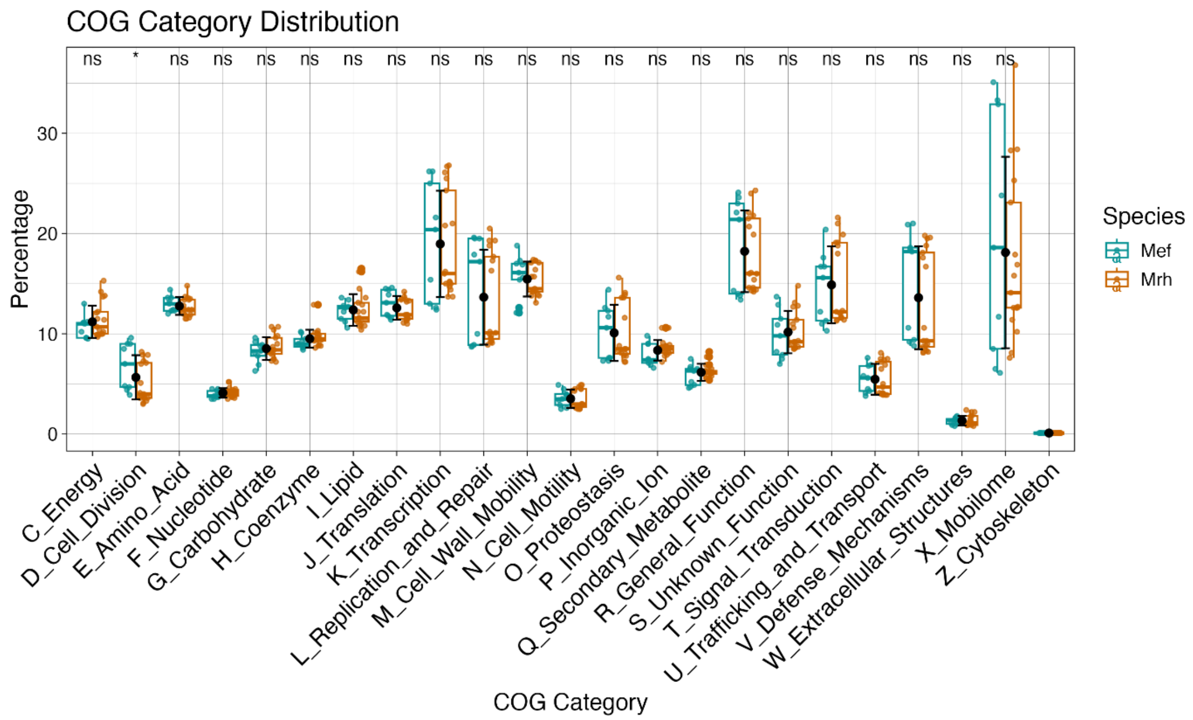

Figure S6. Species wise distribution of genes into functional categories. The red dot indicates the mean of all points, and the red error bar indicates standard deviation. Significance values are indicated for a Wilcoxon test between each category for the two species ($p<0.05$). Mrh, *M. rhizoxinica*; Mef, *M. endofungorum*.

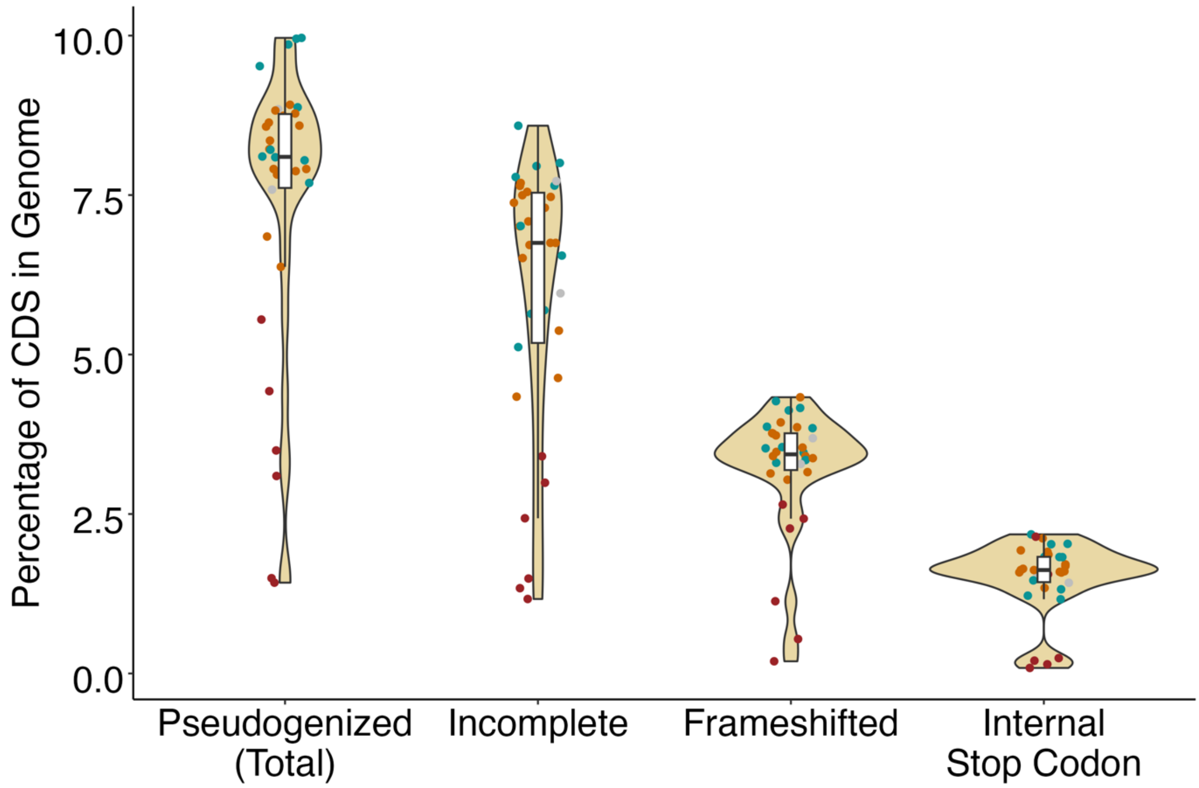

Figure S7. Percentage of the coding DNA sequences (CDS) putatively pseudogenized. Orange dots indicate *M. rhizoxinica,* blue dots indicate *M. endofungorum*, grey dots indicate *Mycetohabitans* spp., and deep red dots indicate representative *Burkholderia* sensu lato spp.

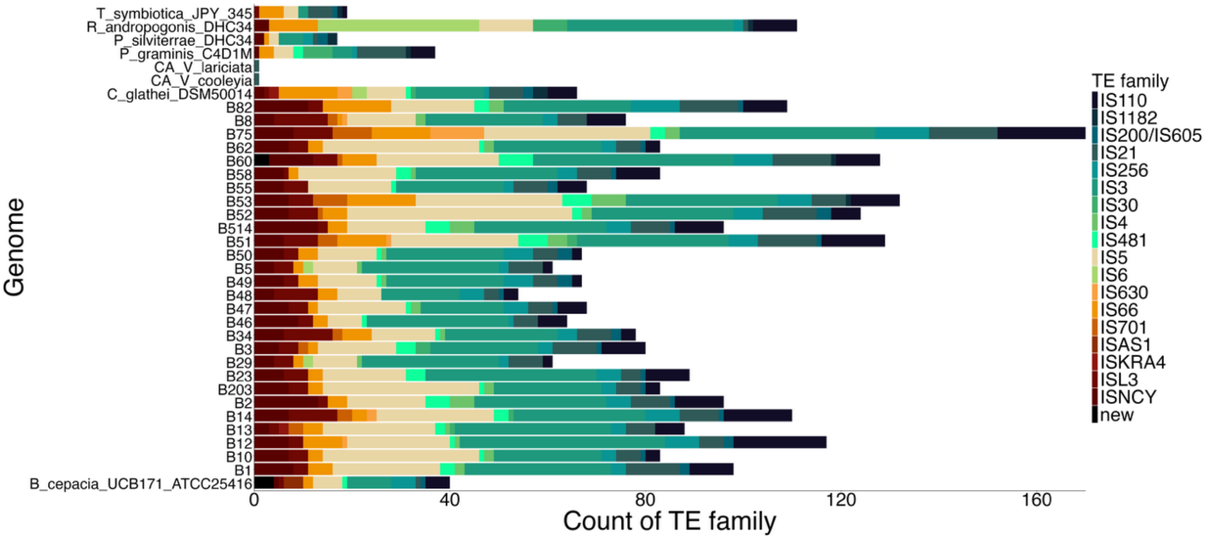

Figure S8. Stacked plot indicating the quantity of transposable elements (TEs) from the indicated families as annotated within each *Mycetohabitans* genome.

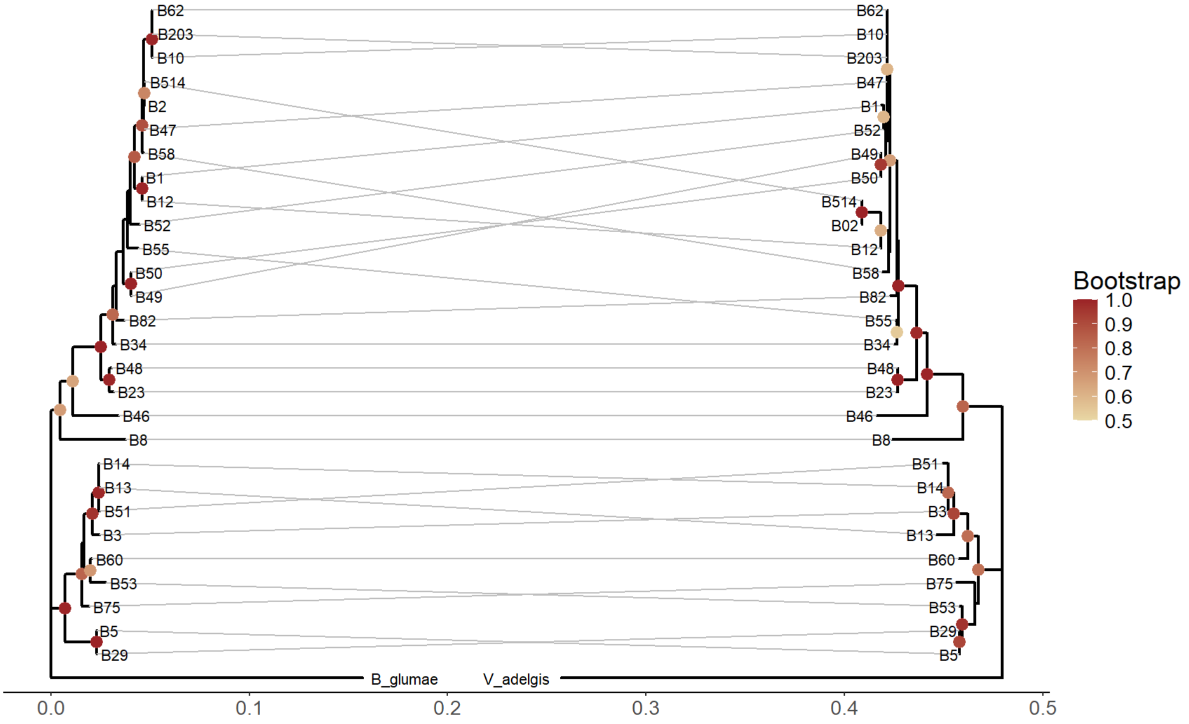

Figure S9. Replication origin -based phylogeny comparison between *Mycetohabitans* chromosomes (L) and chromids (R).

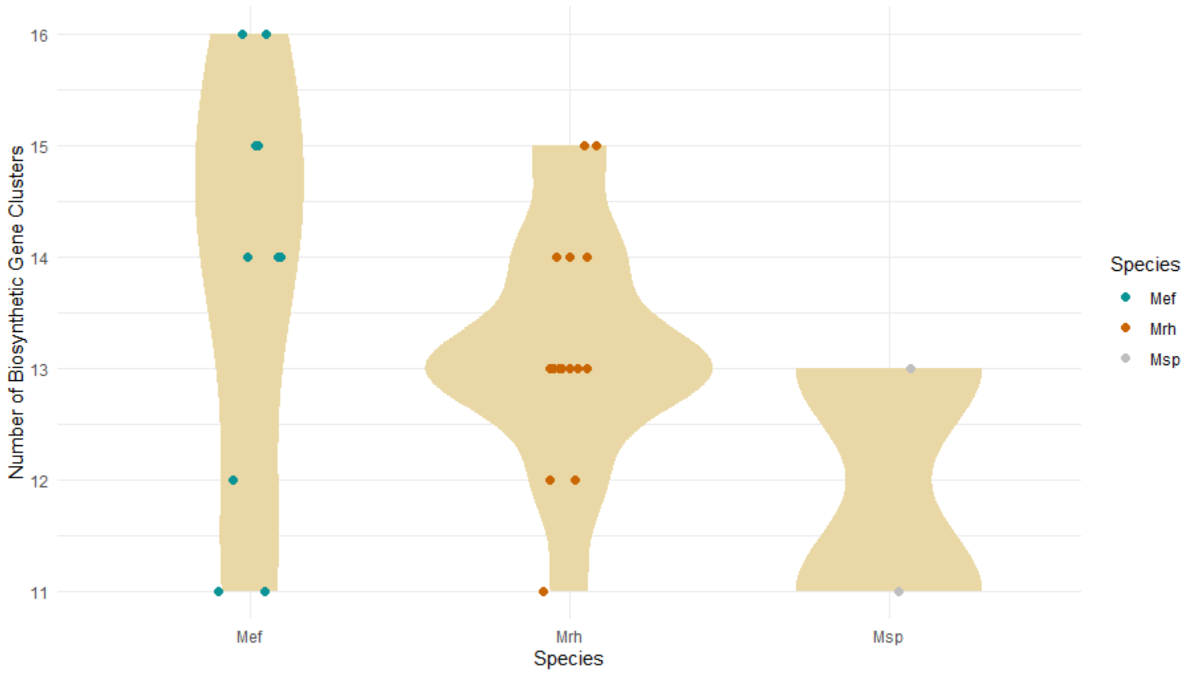

Figure S10. Biosynthetic gene clusters found in all *Mycetohabitans* strains. Msp, *Mycetohabitans* spp.; Mrh, *M. rhizoxinica*; Mef, *M. endofungorum*.

antiSMASH B23

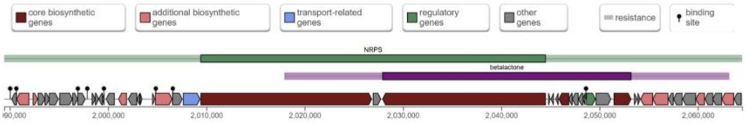

Clinker NRP Group 1

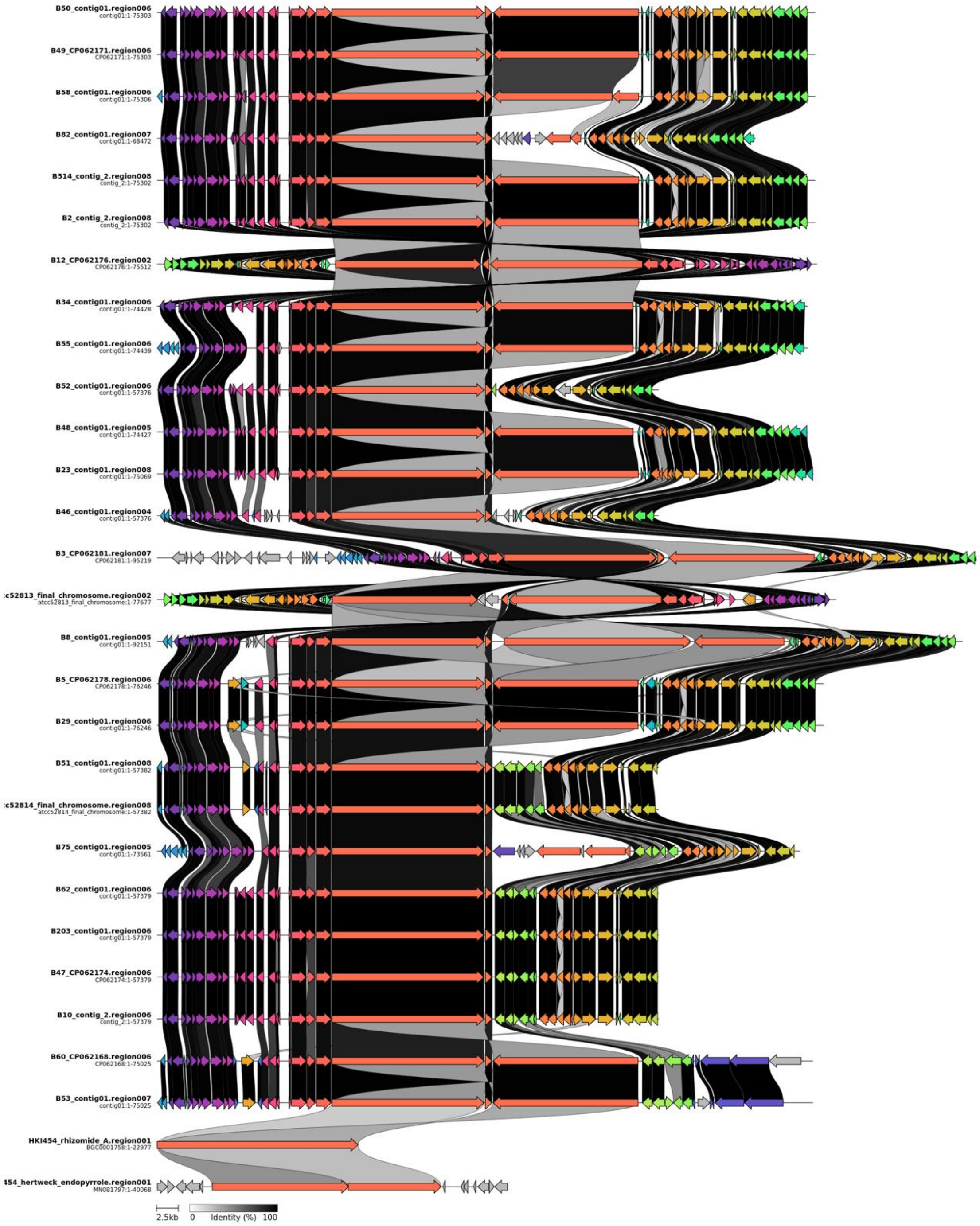

Figure S11. NRP Group 1 gene clusters across *Mycetohabitans* strains showing syntenic conservation and percent identity between each biosynthetic gene in the cluster between strains. Darker connectors indicate higher identity.

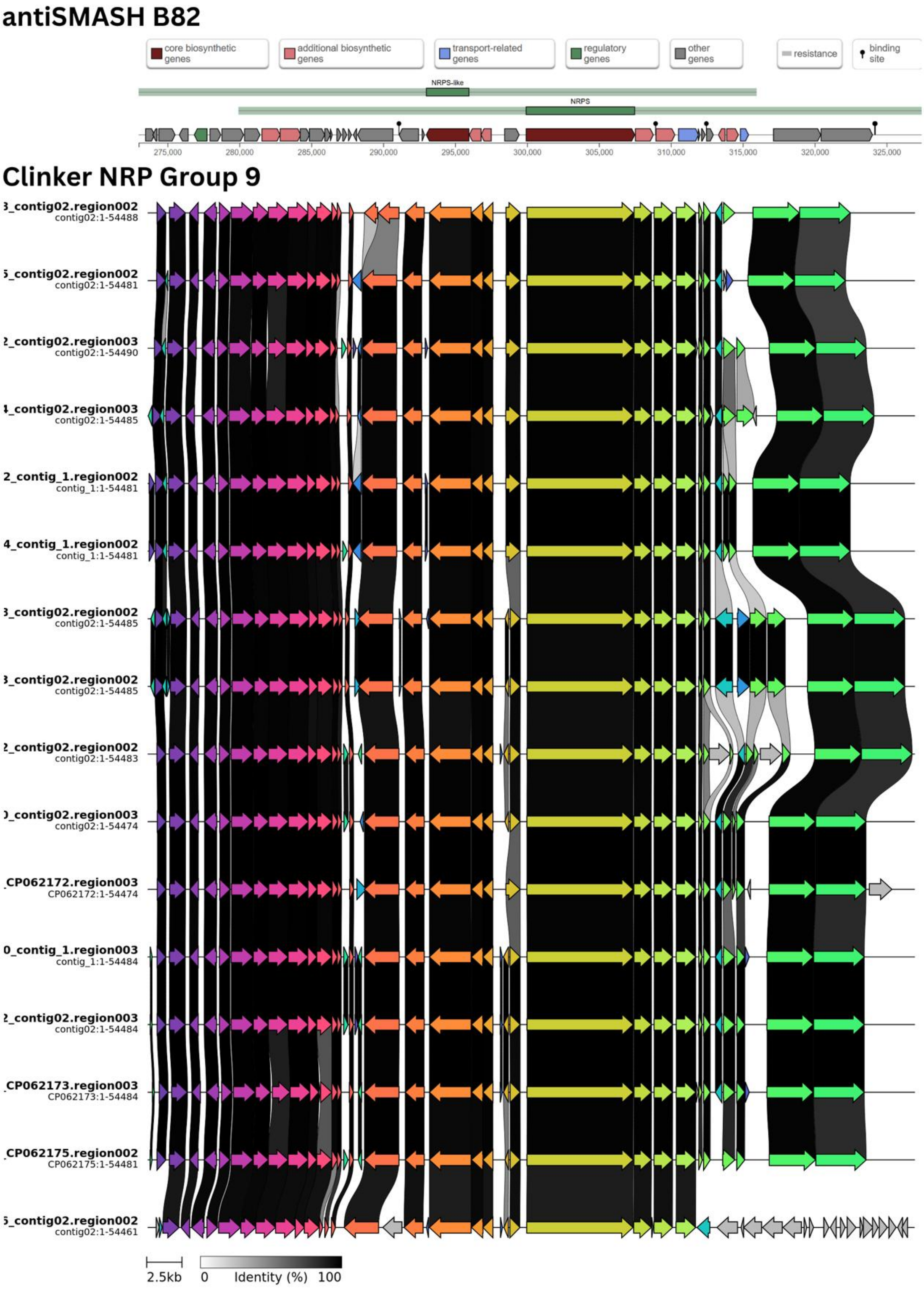

Figure S12. NRP Group 9 gene clusters across *Mycetohabitans* strains showing syntenic conservation and percent identity between each biosynthetic gene in the cluster between strains. Darker connectors indicate higher identity.
